# The Obulamu Mama framework: development of an evidence-informed implementation framework for community obstetric referral systems in rural Uganda

**DOI:** 10.1080/16549716.2026.2749302

**Published:** 2026-10-27

**Authors:** Krista Whitley, Emmanuel Twesige, Andre Craig Christie, Kai Davide Castellarin, Kidus Yohannes, Daniel de Leon, Nada Dualeh, Sellena Chen

**Affiliations:** a School of Global Public Health, New York University, New York, NY, USA; b Department of Emergency Medicine, Kirk Kerkorian School of Medicine at UNLV, Las Vegas, NV, USA; c Faculty of Humanities and Social Sciences, Mountains of the Moon University, Fort Portal City, Uganda; d Trinity Medical Sciences University, Warner Robins, GA, USA; e Department of Psychology, University of Minnesota Twin Cities, Minneapolis, MN, USA; f Life Sciences, University of Nevada, Las Vegas, NV, USA

**Keywords:** Maternal health, obstetric referral, Uganda, health systems strengthening, implementation science

## Abstract

**Background:**

Preventable maternal deaths remain disproportionately concentrated in low- and lower-middle-income countries. In rural Uganda, delayed recognition of obstetric emergencies, transportation barriers, fragmented referral pathways, and inconsistent communication continue to limit timely access to emergency obstetric care.

**Objective:**

To develop an evidence-informed implementation framework for strengthening community obstetric referral systems in rural Uganda.

**Methods:**

We conducted a framework development study using a structured, iterative five-phase process informed by maternal and newborn health evidence, implementation science, systems thinking, international health-system guidance, and Ugandan health policy. Evidence was identified through purposive, targeted searches of PubMed, World Health Organization resources, and Uganda Ministry of Health policy and guidance repositories. Development included problem identification, identification of evidence-informed implementation strategies, preliminary framework development, contextual refinement through in-country review by a Ugandan academic collaborator, and final framework integration.

**Results:**

The Obulamu Mama Framework comprises six interdependent implementation domains: community workforce strengthening; standardized community midwifery resources; closed-loop digital referral communication; coordinated referral networks; continuous quality improvement and supportive supervision; and monitoring, evaluation, and adaptive learning. The domains are designed as complementary components of an integrated maternal referral system aligned with existing community and district health structures.

**Conclusions:**

The framework translates existing evidence and health-systems principles into an implementation roadmap for community obstetric referral systems in rural Uganda. It provides a foundation for feasibility testing, implementation research, and prospective evaluation and may inform adaptation of referral-system strengthening approaches in other resource-constrained settings.

## Paper Context

- **Main findings:** The Obulamu Mama Framework organizes six conceptually interdependent implementation domains into a coordinated approach intended to strengthen community obstetric referral systems in rural Uganda.
- **Added knowledge:** The framework integrates maternal health evidence, implementation science, systems thinking, and Ugandan health-system context into a practical implementation roadmap rather than treating referral interventions as isolated activities.
- **Global health impact for policy and action:** The framework provides a structured basis for adapting, implementing, and evaluating coordinated maternal referral-system strengthening in resource-constrained settings.

## Background

Despite progress since 2000, an estimated 260,000 women died from pregnancy- and childbirth-related complications in 2023, approximately 92% in low- and lower-middle-income countries [1,2]. Most maternal deaths are preventable through timely skilled care, effective referral systems, and high-quality emergency obstetric and newborn care [2–4].

Uganda’s public healthcare system is a hierarchical, decentralized referral system extending from community structures to national referral hospitals. Health Centre II (HC II) facilities provide parish-level outpatient, preventive, and maternal and neonatal services; Health Centre III (HC III) facilities operate at subcounty level and add maternity, delivery, inpatient, and more advanced clinical care [5]. Community Health Extension Workers (CHEWs) are trained parish-level workers introduced under national community health policy, while Village Health Teams (VHTs) remain volunteer community health workers linking households to the formal system [6,7]. Despite progress, preventable maternal mortality remains a major challenge in rural and underserved communities [8–10]. The 2022 Uganda Demographic and Health Survey estimates maternal mortality at 189 deaths per 100,000 live births and neonatal mortality at 22 deaths per 1,000 live births [8]. Geographic isolation, transportation barriers, workforce shortages, delayed recognition, inconsistent communication, and fragmented referral pathways continue to impede timely emergency obstetric care [9,10]. Improving maternal outcomes therefore requires both clinical access and stronger coordination across community, primary-care, and referral levels.

Community-based interventions involving Village Health Teams have improved maternal danger-sign recognition, antenatal care attendance, facility delivery, and timely care-seeking in Uganda, while mobile health interventions have strengthened communication between community and facility providers [11–14]. However, maternal referral systems across sub-Saharan Africa remain constrained by transportation barriers, fragmented communication, weak referral documentation, and poor coordination between sending and receiving facilities [15]. Supportive supervision, digital health interventions, and district-level maternal health programs have strengthened individual components of referral systems, yet these strategies have largely been evaluated in isolation rather than as integrated health-system interventions [16–18]. Collectively, the evidence suggests that effective implementation strategies exist, but important questions remain regarding how they can be systematically organized into a coordinated implementation framework capable of strengthening referral performance across the health system.

Implementation science and systems thinking provide complementary approaches to this challenge. Implementation science addresses how evidence-based practices are adopted, implemented, evaluated, adapted, and sustained and distinguishes implementation from clinical outcomes [19–21]. Systems thinking conceptualizes health systems as interconnected networks in which changes in one component may affect others [22]. Together, these perspectives support frameworks that account for context, component interactions, and continuous learning. Although individual maternal referral interventions have been evaluated, fewer studies have integrated evidence-informed strategies into a coordinated health-system implementation framework.

To address this gap, we conducted an evidence-informed framework development study to develop the Obulamu Mama Framework for strengthening community obstetric referral systems in rural Uganda. Rather than introducing a new clinical intervention, this study synthesizes existing evidence into an integrated implementation framework that organizes complementary strategies across community, district, and other referral health-system levels. Framework development was informed by the Three Delays Framework, systems thinking, implementation science, and contextual refinement by a Ugandan academic collaborator [19–23]. The resulting framework comprises six interdependent implementation domains: community workforce strengthening, standardized community midwifery resources, closed-loop digital referral communication, coordinated referral networks, continuous quality improvement and supportive supervision, and monitoring, evaluation, and adaptive learning. The objective of this study is to describe the framework development process, its conceptual foundations, and the resulting implementation framework intended to guide future adaptation, implementation, and evaluation.

## Methods

### Study design

This framework development study used a structured, iterative process integrating maternal and newborn health evidence, implementation science, systems thinking, health-system guidance, and contextual review by a Ugandan academic collaborator. The Obulamu Mama Framework was the primary output; no intervention or clinical or service-delivery outcomes were evaluated.

Framework development was informed by guidance for developing complex health interventions, which emphasizes consideration of context, programme theory, stakeholder perspectives, interactions among intervention components, and uncertainties requiring future feasibility testing and evaluation [24]. Because the framework was designed to strengthen referral systems across community, referral, and district health-system levels, a systems-thinking approach was adopted to emphasize interactions among implementation strategies rather than treating them as independent interventions [22,24].

### Framework development

Framework development occurred through five iterative phases: (1) problem identification, (2) identification of evidence-informed implementation strategies, (3) preliminary framework development, (4) contextual refinement through in-country academic review, and (5) final framework integration. Although presented sequentially, the process was iterative, with repeated review and revision as evidence, conceptual frameworks, and contextual feedback were incorporated.

The framework was organized around four core functions: identification of obstetric risk, referral coordination, communication across levels of care, and continuous system learning through quality improvement and monitoring.

The five-phase framework development process is summarized in Figure 1.

**Figure 1.**
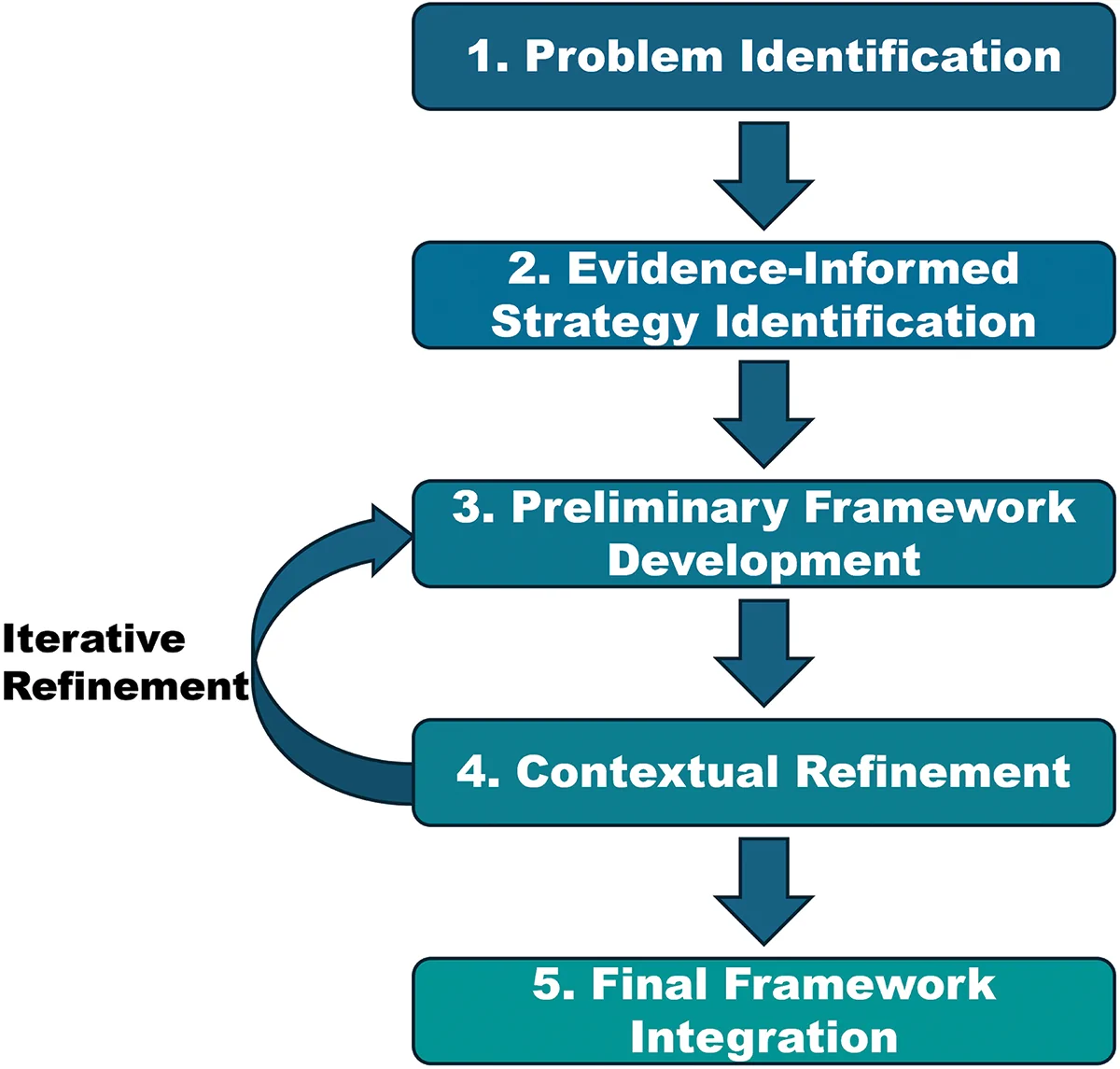
Five-phase framework development process used to develop the Obulamu Mama framework. the process integrated published evidence, conceptual frameworks, and contextual refinement by a Ugandan academic collaborator.

## Phase 1. Problem identification

The objective of Phase 1 was to define the principal health-system barriers contributing to delays in community obstetric referral in rural Uganda. Evidence was synthesized from peer-reviewed literature and authoritative guidance addressing maternal and newborn health, Village Health Teams, emergency obstetric referral, transportation, digital communication, workforce performance, and health-system coordination in Uganda and comparable low-resource settings [8–18].

The Three Delays Framework was used to categorize barriers according to delays in deciding to seek care, reaching an appropriate health facility, and receiving timely, adequate treatment after arrival [23]. Common barriers included limited recognition of maternal and neonatal danger signs, delayed referral decision-making, transportation constraints, fragmented communication between referring and receiving providers, incomplete referral documentation, limited facility preparedness, and inadequate feedback between community and facility levels [11–18,23].

Consistent with systems-thinking principles, these barriers were conceptualized as interacting components of a complex health system rather than independent causes of delayed care. This approach recognized that changes in one component of the referral system may influence performance across other components through interconnected relationships and feedback processes [22]. The outputs of this phase informed the identification and organization of implementation strategies in subsequent phases.

## Phase 2. Identification of evidence-informed implementation strategies

The objective of Phase 2 was to identify implementation strategies that addressed the health-system barriers identified in Phase 1. Evidence identification was purposive and iterative because the objective was framework development rather than a systematic review or meta-analysis. Searches were conducted from 26 January 2026 through 18 August 2026 across PubMed, World Health Organization resources, and Uganda Ministry of Health policy and guidance repositories and incorporated peer-reviewed primary studies, systematic reviews, international guidance, and national maternal and community health policy documents [3,4,6,8–18,22,25].

Search concepts were defined from the referral-system barriers identified in Phase 1. PubMed searches used combinations of terms representing three concept groups: (1) geographic and health-system context (Uganda, sub-Saharan Africa, and low- and middle-income countries); (2) maternal and newborn health (maternal, obstetric, pregnancy, childbirth, and newborn); and (3) referral-system and implementation functions (referral, community health worker, Village Health Team, Community Health Extension Worker, transportation, ambulance, mobile health, mHealth, SMS, communication, supportive supervision, quality improvement, and audit and feedback). Terms within concept groups were combined with OR and concept groups with AND. World Health Organization and Uganda Ministry of Health repositories were searched using the same concepts to identify relevant national policies, implementation guidance, health-system standards, and community and maternal health guidance.

Sources were eligible when they were available in English and comprised primary empirical studies, systematic or scoping reviews, implementation or health-systems studies, or authoritative national or international policy and guidance documents relevant to maternal or newborn referral systems or at least one implementation function represented in the framework. Relevant functions included community-provider capacity, danger-sign recognition and referral initiation, standardized assessment and documentation, referral communication, transportation and coordination, supportive supervision, quality improvement, and monitoring and evaluation. Uganda-specific evidence was prioritized, followed by evidence from sub-Saharan Africa and other low- and middle-income or resource-constrained settings when relevant Ugandan evidence was limited. Foundational implementation-science, health-systems, and conceptual literature was eligible irrespective of geographic setting when it provided an established theoretical or methodological basis for framework development or future evaluation.

Sources were excluded when they did not address maternal or newborn care or an implementation function relevant to the referral pathway; focused exclusively on clinical treatment without referral or health-system implementation implications; were opinion pieces or commentaries without substantive empirical, methodological, or policy content; duplicated another retrieved source; or were unavailable in English. No publication-date restriction was imposed because foundational literature, including the Three Delays Framework, was required for the conceptual development of the framework.

Peer-reviewed records were assessed for relevance to the eligibility criteria, and potentially relevant sources were reviewed in full. Policy and guidance documents were assessed for relevance to Uganda’s community health structure, maternal and newborn care, referral systems, or one or more framework implementation functions. Eligible sources were then classified according to the implementation function or functions they informed; a source could contribute to more than one function when its findings addressed multiple components of the referral pathway. Final retention required that a source contribute empirical evidence, authoritative guidance, contextual information, or a conceptual basis for at least one framework domain.

Because evidence identification was purposive and iterative rather than a prospectively registered systematic review, no formal screening log was maintained and record-level screening counts cannot be reconstructed reliably. Exact database-specific historical search strings were not prospectively archived. The reported terms, concept groups, Boolean structure, sources, and search dates therefore document the approach used but are not a verbatim reconstruction of every historical query.

No formal study-level risk-of-bias or methodological-quality appraisal tool was applied during the original framework-development process. Sources were used according to their role in framework development rather than treated as equivalent evidence of effectiveness: empirical studies informed observed referral and implementation functions; systematic and scoping reviews informed recurring evidence across studies; national and international policy or guidance documents informed recommended health-system structures and standards; and conceptual or implementation-science sources informed programme theory and future evaluation. Relevance, geographic and health-system applicability, and source type informed interpretation. Evidence that was limited, heterogeneous, or drawn from settings outside Uganda was used to identify potentially relevant implementation functions but was not interpreted as demonstrating effectiveness in rural Uganda. Inclusion of a strategy in the framework therefore indicates evidence-informed relevance, not a formal judgment of evidence certainty or demonstrated effect.

Evidence extraction and classification were organized around the implementation functions identified during framework development rather than a prospectively specified qualitative coding instrument. The two corresponding authors developed the initial six-domain structure and mapped evidence to the implementation function or functions it addressed. When a source informed more than one operational function, it was permitted to contribute to more than one domain rather than being forced into a single mutually exclusive category. The remaining authors subsequently reviewed the supporting evidence and the proposed strategy-to-domain assignments. Proposed changes or disagreements were discussed by the author group and incorporated by consensus. This process served as the consistency check for domain classification; independent duplicate coding and a statistical measure of inter-reviewer agreement were not performed.

Implementation strategies were extracted according to their primary function within the maternal referral continuum and iteratively grouped into thematic categories representing common operational functions. Evidence supporting community-provider training, competency assessment, supervision, and integration informed the community workforce strengthening domain [11–13,17,25]. Evidence related to standardized assessment and initial stabilization informed the standardized community midwifery resources domain [3,4]. Studies of mobile consultation and digital health informed the closed-loop digital referral communication domain [13,14,16]. Evidence addressing transportation, referral quality, facility coordination, and multisectoral maternal health programmes informed the coordinated referral networks domain [12,15,18]. Evidence supporting supportive supervision, quality improvement, and implementation evaluation informed the continuous quality improvement and supportive supervision and monitoring, evaluation, and adaptive learning domains [17,18,20].

Distinct operational categories recurring across evidence sources were retained, resulting in six preliminary evidence-informed implementation domains.

## Phase 3. Development of the preliminary framework

The objective of Phase 3 was to synthesize the six preliminary implementation domains into a unified implementation framework. Framework development was informed by the Three Delays Framework, systems thinking, and implementation science as complementary conceptual foundations [19–23]. The Three Delays Framework linked implementation strategies to recognized delays in maternal care, systems thinking guided consideration of interactions among community providers, referral facilities, transportation systems, communication infrastructure, district leadership, and feedback processes, and implementation science informed consideration of feasibility, acceptability, adoption, fidelity, and sustainability as future implementation outcomes [19–23].

The preliminary framework was organized so that each implementation domain contributed to one or more of the four core health-system functions. Rather than functioning as independent interventions, the domains were designed to operate as complementary components of a coordinated referral system. Community workforce strengthening was intended to support early recognition and referral initiation; standardized community midwifery resources were intended to promote consistent assessment and communication; closed-loop digital referral communication was intended to strengthen coordination between referring and receiving providers; coordinated referral networks were intended to address transportation and organizational linkages; and continuous quality improvement, supportive supervision, and monitoring were intended to support ongoing system learning and adaptation. The proposed interdependence of the six domains is conceptual rather than an empirically demonstrated property of the framework. It derives from the Three Delays Framework and systems thinking, which conceptualize referral performance as the product of interacting patient-, community-, transport-, communication-, facility-, and organizational processes [19,22]. The present study does not establish that all six domains are necessary or sufficient for effectiveness.

Framework development also incorporated guidance for developing complex health interventions by explicitly considering interactions among implementation components, contextual influences, programme theory, and future feasibility testing and evaluation [24].

## Phase 4. Contextual refinement through in-country academic review

The objective of Phase 4 was to evaluate the contextual appropriateness of the preliminary framework for implementation within the Ugandan health system. Contextual refinement was conducted through iterative in-country review by one Ugandan academic collaborator and co-corresponding author, Emmanuel Twesige, Assistant Lecturer in Social Sciences in the Faculty of Humanities and Social Sciences at Mountains of the Moon University in Fort Portal, Uganda. His disciplinary perspective in social sciences and knowledge of local organizational structures and the Ugandan health-system context informed review of terminology, stakeholder roles, referral pathways, transportation constraints, communication processes, and alignment with Uganda’s community health structure, including Community Health Extension Workers (CHEWs) and Village Health Teams (VHTs). Feedback was incorporated iteratively into successive versions of the framework. This process was intended to improve contextual appropriateness rather than establish formal expert consensus, constitute stakeholder engagement, or validate the framework.

Uganda’s community health structure positions CHEWs as trained parish-level workers intended to strengthen community-level primary health care, while VHTs remain volunteer community health workers linking households with formal health services [6,7]. The VHT strategy emphasizes community ownership, coordination, capacity building, referral, and integration with district health structures [6]. World Health Organization guidance similarly recommends adapting community health worker programmes to local health-system contexts through appropriate education, supervision, integration, and community engagement [25].

Recommendations from the contextual review informed refinement of framework terminology, referral pathways, implementation assumptions, and stakeholder roles with the aim of improving compatibility with existing community health infrastructure.

## Phase 5. Final framework integration

Phase 5 integrated contextual feedback through iterative revision of domains, referral pathways, terminology, and implementation assumptions to improve compatibility with Uganda’s community and district health systems.

The final Obulamu Mama Framework comprised six interdependent implementation domains: (1) community workforce strengthening, (2) standardized community midwifery resources, (3) closed-loop digital referral communication, (4) coordinated referral networks, (5) continuous quality improvement and supportive supervision, and (6) monitoring, evaluation, and adaptive learning. Each domain was linked to one or more core health-system functions and designed to operate as part of a coordinated referral system rather than as an independent intervention. The completed framework represented the primary output of this framework development study. The evidence base, major source categories, and primary implementation functions informing each domain are summarized in Table 1.

**Table 1.** Evidence informing each implementation domain of the Obulamu Mama framework.

Domain	Major Evidence Sources	Primary Implementation Function
Community Workforce Strengthening	Kananura et al. [12]; Mangwi Ayiasi et al. [13]; WHO CHW Guideline [25]; Uganda VHT Strategy [6]; Uganda CHEW Policy [7]	Risk recognition and referral initiation
Standardized Community Midwifery Resources	WHO Quality Standards [4]; Ameyaw et al. [15]; WHO Intrapartum Care Guideline [26]	Standardized assessment, documentation, and referral preparation
Closed-Loop Digital Referral Communication	Mangwi Ayiasi et al. [13,14]; Knop et al. [16]; Lee et al. [27]	Referral communication, provider consultation, and information transfer
Coordinated Referral Networks	Ameyaw et al. [15]; Serbanescu et al. [18]; Thaddeus & Maine [23]; Hussein et al. [28]	Referral coordination, transportation, and facility linkage
Continuous Quality Improvement and Supportive Supervision	Deussom et al. [17]; WHO CHW Guideline [25]; Ivers et al. [29]	Supervision, audit and feedback, and performance improvement
Monitoring, Evaluation, and Adaptive Learning	De Savigny & Adam [22]; Damschroder et al. [19]; Proctor et al. [20]; Skivington et al. [24]	Monitoring implementation, evaluation outcomes, and adaptive learning

Abbreviations: CHEW, Community Health Extension Worker; CHW, Community Health Worker; VHT, Village Health Team; WHO, World Health Organization.

## Ethics statement

This study synthesized published literature, publicly available policy and guidance documents, and scholarly input from the author team. No research participants, identifiable personal information, patient records, or animal subjects were involved; institutional review board approval and informed consent were therefore not required.

## Results

### Development of the Obulamu Mama Framework

Framework development resulted in the Obulamu Mama Framework, an evidence-informed implementation framework for strengthening community obstetric referral systems in rural Uganda (Figure 2). The framework comprises six interdependent implementation domains: (1) community workforce strengthening, (2) standardized community midwifery resources, (3) closed-loop digital referral communication, (4) coordinated referral networks, (5) continuous quality improvement and supportive supervision, and (6) monitoring, evaluation, and adaptive learning. Together, these domains are designed to address key barriers to timely recognition, referral, transportation, communication, and coordination of obstetric emergencies.

**Figure 2.**
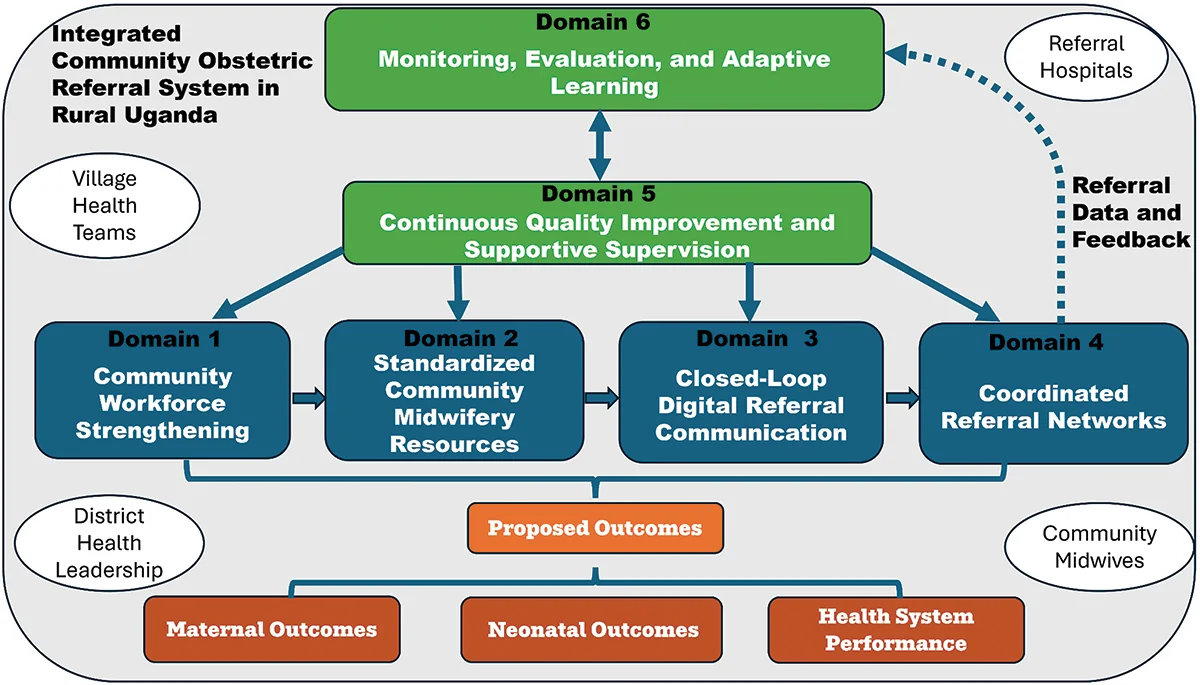
Proposed Obulamu Mama framework for an integrated community obstetric referral system in rural Uganda.

The framework was organized around the principle that referral performance depends on interactions among community providers, referral facilities, transportation systems, communication networks, and district health leadership. Accordingly, each implementation domain is intended to support one or more core health-system functions while reinforcing coordination across the referral pathway. Rather than functioning as independent interventions, the domains were designed to operate as complementary components of an integrated maternal referral system built upon Uganda’s existing community health infrastructure.

### Domain 1. Community Workforce Strengthening

The Community Workforce Strengthening domain focuses on preparing community-based providers to recognize obstetric complications and initiate timely referral. Evidence indicates that community health workers and Uganda’s Village Health Teams improve maternal and newborn health knowledge, promote skilled birth attendance, facilitate earlier recognition of obstetric danger signs, and support timely referral when appropriately trained and integrated within formal health systems [8–10,21].

Within the Obulamu Mama Framework, this domain incorporates competency-based education for community midwives, Community Health Extension Workers, and Village Health Team members, emphasizing recognition of maternal and neonatal danger signs, identification of high-risk pregnancies, standardized referral decision-making, initial stabilization within the scope of community practice, and effective referral communication. Workforce strengthening also includes supportive supervision, periodic competency assessment, continuing professional development, and integration within existing district health systems, consistent with World Health Organization guidance [25]. Ongoing supervision and performance feedback have been associated with improved competency retention, implementation fidelity, provider confidence, and quality of care compared with one-time training alone [17,25]. Collectively, these components are intended to strengthen early recognition of obstetric emergencies and promote timely, consistent referral initiation.

### Domain 2. Standardized Community Midwifery Resources

The Standardized Community Midwifery Resources domain provides clinical resources to support maternal assessment, initial stabilization, and referral within community settings. Standardized assessment tools, referral documentation, and clinical protocols are core components of quality maternal care and support consistent evidence-based practice, communication, and recognition of obstetric emergencies [3,4,26]. Standardized referral documentation also supports continuity of care by facilitating the transfer of essential clinical information between referring and receiving providers [4,15].

Within the Obulamu Mama Framework, this domain includes structured assessment guides, standardized referral documentation, essential equipment appropriate to the scope of community practice, and decision-support resources to support consistent identification of high-risk pregnancies and obstetric complications. These resources are intended to improve the consistency of assessment, stabilization when appropriate, and communication before referral rather than expand definitive obstetric care at the community level. Standardization is also intended to reduce variation in clinical practice and support continuity of information across the referral network [4,15]. Collectively, these components are intended to support continuity of care from community assessment through referral to higher-level facilities.

### Domain 3. Closed-Loop Digital Referral Communication

The Closed-Loop Digital Referral Communication domain establishes a structured communication pathway linking community providers with receiving health facilities through a standardized SMS-based referral system. Evidence indicates that timely communication between referring and receiving providers improves referral coordination, continuity of care, and preparedness for obstetric emergencies, while reducing delays associated with fragmented communication and incomplete transfer of clinical information [14–16,27].

Within the Obulamu Mama Framework, referral notifications are transmitted electronically from community providers to receiving facilities using a standardized SMS platform. Receiving facilities acknowledge referrals, confirm receipt of essential clinical information, and maintain communication throughout the referral process. This closed-loop approach promotes accountability, coordination, and continuity of care across referral levels.

In addition to supporting individual referrals, the communication platform generates standardized referral data that can be incorporated into routine monitoring, quality improvement, and health system management [16,20]. These data support evaluation of communication performance, referral completion, and operational bottlenecks while informing continuous system improvement. The communication domain is designed to complement existing referral relationships and operate alongside the other implementation domains to strengthen coordination across the maternal referral pathway.

### Domain 4. Coordinated Referral Networks

The Coordinated Referral Networks domain strengthens coordination among community providers, transportation resources, district health leadership, and referral hospitals. Evidence indicates that fragmented referral pathways, transportation delays, inconsistent communication, and weak coordination between referring and receiving facilities contribute to delays in accessing emergency obstetric care and may adversely affect maternal outcomes in low-resource settings [15,18,23,28,30].

Within the Obulamu Mama Framework, coordinated referral networks are supported through standardized referral protocols, clearly defined communication pathways, designated referral linkages between community and facility providers, and routine coordination among district health stakeholders. Transportation planning and community-supported transport mechanisms are incorporated as integral components of referral coordination to improve timely access to comprehensive emergency obstetric care [23,28,30].

By integrating transportation planning, organizational coordination, and standardized referral processes, this domain is intended to strengthen continuity of care, clarify referral responsibilities, and improve coordination across the maternal referral pathway. Evidence from district-level maternal health initiatives demonstrates that coordinated, multisectoral approaches can strengthen maternal health service delivery and improve maternal health outcomes [18].

### Domain 5. Continuous Quality Improvement and Supportive Supervision

The Continuous Quality Improvement and Supportive Supervision domain embeds quality improvement and supportive supervision within routine maternal referral operations. Evidence indicates that supportive supervision strengthens health worker performance, implementation fidelity, competency retention, and service quality compared with one-time educational interventions [17,25]. Audit and feedback have also been associated with improvements in professional practice and healthcare delivery, particularly when feedback is delivered regularly and accompanied by clear action plans [29].

Within the Obulamu Mama Framework, this domain is operationalized through structured supervisory visits, multidisciplinary case review, routine audit and feedback, and collaborative performance review across the referral pathway. These activities are intended to identify operational barriers, reinforce standardized referral practices, promote collaborative problem-solving, and support continuous refinement of referral processes among community providers, referral facilities, and district health leadership.

By embedding supportive supervision and continuous quality improvement within routine district operations, this domain is intended to promote organizational learning, strengthen implementation fidelity, and support continuous improvement of the maternal referral system [19–21].

### Domain 6. Monitoring, Evaluation, and Adaptive Learning

The Monitoring, Evaluation, and Adaptive Learning domain is designed to provide a structured approach for assessing implementation and service-delivery performance across the Obulamu Mama Framework. Evidence from implementation science and health systems strengthening emphasizes the routine use of monitoring data to evaluate implementation, identify barriers, support decision-making, and guide continuous improvement [19–22].

Within the Obulamu Mama Framework, monitoring and evaluation incorporate routine collection and review of implementation and service-delivery indicators. Proposed implementation measures include provider competency following training, adherence to standardized referral procedures, utilization of referral documentation, referral response times, facility acknowledgment of referrals, referral completion, and participation in supportive supervision activities. Service-delivery indicators include referral timeliness, selected maternal and neonatal outcomes, and overall referral system performance. Together, these measures provide district health leadership with actionable information to support evidence-informed decision-making and future evaluation.

For prospective evaluation, referral response time is defined as minutes from transmission of a referral message to acknowledgment by the designated receiving facility and would be obtained from time-stamped digital communication logs. Referral timeliness is defined as elapsed minutes from the documented referral decision to arrival at the designated receiving facility, using referral records linked to facility arrival records. Referral completion is the proportion of initiated referrals documented as arriving at the intended receiving facility. Provider competency is the percentage of prespecified knowledge or skills-assessment items successfully completed, measured before training, immediately after training, and at prespecified follow-up intervals. Implementation fidelity is the proportion of required referral-process components completed as specified, assessed using referral records, digital logs, and structured audit or supervision checklists. Facility acknowledgment is the proportion of transmitted referrals receiving a documented response from the designated facility. Continuous time measures would be summarized using means and standard deviations or medians and interquartile ranges according to distribution; binary outcomes would be reported as proportions with confidence intervals. Prospective comparative studies could use multivariable generalized linear or mixed-effects models, as appropriate to the outcome and clustered health-system structure, and time-to-event methods for referral intervals when censoring or variable follow-up is relevant. Baseline, early implementation, and prespecified follow-up measurement periods should be defined prospectively according to the implementation design. These are proposed evaluation specifications and were not applied in the present framework-development study. The proposed implementation and service-delivery outcomes for each framework domain, along with their corresponding data sources for prospective evaluation, are summarized in Table 2.

**Table 2.** Proposed implementation and service-delivery outcomes for the Obulamu Mama framework.

Domain	Proposed Implementation Outcomes	Proposed Service Delivery Outcomes
Community Workforce Strengthening	Provider competency; fidelity to referral procedures	Timeliness and appropriateness of referral initiation
Standardized Community Midwifery Resources	Utilization of standardized assessment and referral documentation; protocol adherence	Completeness and quality of referral documentation; continuity of clinical information
Closed-Loop Digital Referral Communication	Adoption and use of the referral system; facility acknowledgment; response time	Timeliness of referral communication; receiving facility preparedness
Coordinated Referral Networks	Adherence to referral protocols; referral completion	Transfer timeliness; referral coordination and completion
Continuous Quality Improvement and Supportive Supervision	Participation in supportive supervision; audit and feedback completion; implementation fidelity	Referral quality and identification of recurring system barriers
Monitoring, Evaluation, and Adaptive Learning	Data completeness; monitoring participation; use of data for adaptation	Referral system performance and continuous system improvement

Consistent with implementation science, the framework distinguishes implementation outcomes from clinical outcomes by proposing measures such as acceptability, appropriateness, adoption, feasibility, fidelity, penetration, and sustainability alongside maternal referral performance [20]. Adaptive learning would be supported through regular review of monitoring data, multidisciplinary reflection, and iterative refinement of implementation activities. These proposed measures and feedback processes are hypotheses for prospective implementation testing and were not evaluated in the current study.

### Integrated health systems framework

The Obulamu Mama Framework is intentionally developed as an integrated implementation framework rather than a collection of independent interventions. Although each implementation domain addresses a distinct function within the maternal referral pathway, the framework is organized around the principle that referral performance depends on coordinated interactions among community providers, referral facilities, transportation systems, communication networks, district leadership, and continuous feedback processes [22].

The six domains are hypothesized to function as complementary components, but this study does not establish that all are necessary or equally influential. Future studies should test individual and combined contributions using multivariable or multilevel models of domain exposure or implementation intensity, prespecified interaction terms, and mediation or path analysis. Where feasible, factorial or sequential multiple-assignment designs could evaluate component combinations and empirically test the framework’s proposed interdependence.

The framework aligns with contemporary principles of health systems strengthening and implementation science by integrating complementary implementation strategies within routine health system practice rather than emphasizing isolated interventions [3,19–22]. Accordingly, the Obulamu Mama Framework is intended to serve as an adaptable implementation roadmap that can be tailored to local resources, organizational structures, and implementation contexts while preserving coordinated system-wide functioning.

## Discussion

This study developed the Obulamu Mama Framework by integrating maternal health evidence, implementation science, systems thinking, and health-system guidance into a coordinated model for community obstetric referral in rural Uganda. Its principal contribution is organizing evidence-informed strategies for future implementation, evaluation, and adaptation rather than proposing a new clinical intervention.

Previous maternal health initiatives have demonstrated improvements through community health worker training, Village Health Team strengthening, digital communication, emergency transportation, supportive supervision, and facility-based quality improvement. Many of these strategies, however, have been evaluated individually despite referral systems functioning as interconnected health-system processes [11–20,22]. Recent mixed-methods evidence from a multilevel referral system further illustrates that referral performance is shaped by interacting facility capacity, human resources, equipment availability, communication, and coordination across levels of care [31]. A complementary health-system perspective emphasizes that workforce roles extend beyond individual clinical tasks to responsibilities toward patients, communities, professional practice, and broader system strengthening in low- and middle-income settings [32]. The Obulamu Mama Framework is designed to address this gap by organizing complementary implementation strategies into six conceptually interdependent implementation domains intended to support coordinated referral-system strengthening rather than isolated changes in individual components of care [22].

The framework builds on Uganda’s existing Village Health Teams, district referral pathways, and national community health policies rather than creating parallel structures [6,24,25]. Contextual refinement by a Ugandan academic collaborator aimed to improve applicability and alignment with existing structures, not establish formal consensus or validate the framework.

Although developed for rural Uganda, the implementation functions addressed by the framework – including community-level risk recognition, referral communication, transportation coordination, supportive supervision, and adaptive learning – reflect recurrent challenges in maternal referral systems across many resource-constrained settings. The framework is therefore intended to support context-specific adaptation rather than direct transfer. Prospective testing should examine which components require modification across differing community health workforce models, referral structures, transportation systems, digital infrastructure, and governance arrangements.

## Relationship to existing frameworks

The Obulamu Mama Framework was developed to complement existing conceptual and implementation science frameworks rather than replace them. The Three Delays Framework remains one of the most influential models for understanding barriers to maternal survival by identifying delays in deciding to seek care, reaching an appropriate health facility, and receiving definitive treatment [23]. However, while the Three Delays Framework identifies where barriers occur, it does not specify how evidence-informed implementation strategies can be organized within routine health-system practice. The Obulamu Mama Framework extends this conceptual foundation by translating recognized referral barriers into coordinated implementation domains that strengthen communication, workforce capacity, referral coordination, and continuous system learning. The relationship between the six implementation domains and the Three Delays Framework is summarized in Table 3.

**Table 3.** Mapping of the Obulamu Mama framework implementation domains to the Three Delays framework.

Domain	Delay 1: Deciding to Seek Care	Delay 2: Reaching Care	Delay 3: Receiving Adequate Care
Community Workforce Strengthening	✔		
Standardized Community Midwifery Resources	✔		✔
Closed-Loop Digital Referral Communication		✔	✔
Coordinated Referral Networks		✔	✔
CQI and Supportive Supervision	✔	✔	✔
Monitoring, Evaluation, and Adaptive Learning	✔	✔	✔

Similarly, the framework operationalizes principles of systems thinking by integrating workforce development, standardized clinical resources, communication systems, referral coordination, supportive supervision, and monitoring within a coordinated implementation model for maternal referral systems rather than addressing these components independently [22].

Established implementation science frameworks such as the Consolidated Framework for Implementation Research (CFIR) provide structured approaches for examining determinants that influence implementation across multiple contextual domains [19–21]. The Obulamu Mama Framework serves a complementary function by organizing evidence-informed maternal referral strategies into an operational structure for coordinated implementation within district health systems. Established implementation science frameworks and outcome measures can subsequently be applied to evaluate determinants, implementation processes, and outcomes during prospective testing of the framework.

The Obulamu Mama Framework also expands upon community health worker and Village Health Team models by recognizing that workforce capacity alone is insufficient to strengthen maternal referral performance. Effective referral systems also depend on coordinated communication, transportation, standardized clinical resources, supportive supervision, governance, and continuous quality improvement. By integrating these complementary components, the Obulamu Mama Framework adopts a systems-oriented approach that reflects the complexity of maternal referral systems in rural, resource-constrained settings.

The Obulamu Mama Framework therefore complements existing theory by organizing evidence, implementation science, and health-systems principles for future adaptation, implementation, evaluation, and scale-up.

## Strengths and limitations

Strengths include integration of maternal health evidence, implementation science, systems thinking, and community health systems; in-country contextual review by a Ugandan academic collaborator; linkage of strategies to measurable implementation and service-delivery outcomes; and alignment with existing community and district health structures.

Several limitations should also be considered. The evidence review was purposive rather than systematic and therefore may not have identified all relevant maternal referral interventions or implementation strategies. A formal study-level risk-of-bias or methodological-quality appraisal was not conducted, and evidence from different source types was not graded for certainty. Independent duplicate coding and statistical inter-reviewer agreement were also not performed; instead, evidence classification and domain assignments were reviewed by the author group and resolved through consensus. The framework development process did not include formal stakeholder consensus methods, such as Delphi procedures, or prospective validation with health workers, district health officials, pregnant women, or community members. Contextual refinement was informed by one Ugandan academic collaborator and co-corresponding author but did not constitute formal stakeholder engagement or framework validation. Additionally, because evidence informing individual framework domains was drawn from heterogeneous studies conducted across Uganda and other low-resource settings, the effectiveness of individual strategies cannot be assumed to transfer directly to all rural Ugandan contexts. Inclusion of an implementation strategy within the framework should therefore be interpreted as evidence-informed relevance to a proposed health-system function rather than proof of effectiveness.

Accordingly, the feasibility, acceptability, appropriateness, and effectiveness of the Obulamu Mama Framework remain to be established through prospective implementation and evaluation. The current study collected no primary outcome data and provides no quantitative estimate of framework effectiveness. Future research should incorporate broader stakeholder engagement, including Village Health Teams, midwives, referral facility staff, district health leaders, pregnant women, families, transport providers, and community representatives, to further refine the framework and assess its implementation within diverse rural Ugandan settings. Established implementation evaluation approaches such as the RE-AIM framework may provide a structured approach for assessing reach, effectiveness, adoption, implementation, and maintenance during prospective testing and scale-up [33].

## Conclusion

The Obulamu Mama Framework integrates maternal health evidence, implementation science, systems thinking, and Ugandan contextual refinement into six conceptually interdependent domains for coordinated community obstetric referral-system strengthening.

The framework translates existing evidence and conceptual models into an operational roadmap emphasizing coordinated workforce development, standardized resources, communication, referral coordination, quality improvement, and continuous learning while remaining adaptable to local contexts.

The framework has not undergone implementation or formal evaluation; this study provides no quantitative evidence of improved referral or clinical outcomes. Prospective research should assess implementation outcomes – including acceptability, appropriateness, adoption, feasibility, fidelity, penetration, sustainability, and cost – alongside referral, maternal and neonatal, and health-system outcomes.

Strengthening referral systems remains essential to achieving the Sustainable Development Goals and the World Health Organization’s Ending Preventable Maternal Mortality targets [3]. The Obulamu Mama Framework provides a structured foundation for future implementation research and offers an adaptable approach that may support maternal referral system strengthening in Uganda and other resource-constrained settings.

## Data Availability

No primary research data were generated or analyzed in this study. The evidence used to develop the framework was derived from published literature and publicly available policy and guidance documents cited in the manuscript. All information supporting the framework development process is contained within the manuscript and its cited sources.
